# Spontaneous Resolution of a Pseudoaneurysm at the Anastomosis Site of Coronary Artery Bypass Graft: A Case Report

**DOI:** 10.7759/cureus.77878

**Published:** 2025-01-23

**Authors:** Tsukasa Miyatake, Taro Minamida, Masaya Watanabe, Noriyoshi Kato, Izumi Yoshida

**Affiliations:** 1 Department of Cardiovascular Surgery, Hokko Memorial Hospital, Sapporo, JPN; 2 Department of Cardiovascular Surgery, Hokkaido University Hospital, Sapporo, JPN; 3 Department of Cardiology, Hokko Memorial Hospital, Sapporo, JPN

**Keywords:** circumflex artery, coronary artery bypass grafting, internal mammary artery, pseudoaneurysm, spontaneous resolution of a pseudoaneurysm

## Abstract

Pseudoaneurysms at the coronary artery bypass graft (CABG) anastomosis site are rare. Here, we report a rare case of spontaneous resolution of a pseudoaneurysm at the anastomosis site developed soon after CABG. A 66-year-old man was referred to our hospital because of chest pain with paroxysmal atrial fibrillation. Pulmonary vein isolation (PVI), atrial appendage closure, and CABG were performed under cardiopulmonary bypass. Contrast computed tomography (CT) on postoperative day (POD) 5 revealed a pseudoaneurysm at the anastomosis of the left internal mammary artery (LIMA) to the circumflex artery. However, the pseudoaneurysm spontaneously resolved after three days. Given the possibility of rupture, surgical or interventional treatment would generally be the first choice. Exceptionally, observation for days to weeks may be one of the management strategies in patients with a small pseudoaneurysm, stable vital signs, no symptoms, discontinuable antithrombotic medications, a short time after CABG, or a pseudoaneurysm at the anastomosis site. The findings in this case may serve as a reference for the determination of treatment strategies for similar cases in the future.

## Introduction

Few cases of pseudoaneurysms following coronary artery bypass grafting (CABG) have been reported [1, 2]. If not diagnosed promptly, it can lead to serious complications [1]. Pseudoaneurysms at the anastomosis site are particularly rare, possibly because they are treated early owing to bleeding during or immediately after surgery. We incidentally discovered a pseudoaneurysm at the anastomosis of the left internal mammary artery (LIMA) to the circumflex artery on contrast computed tomography (CT) five days after CABG. However, it spontaneously resolved within three days. Here we report the findings in this rare case.

## Case presentation

**Figure 1:**
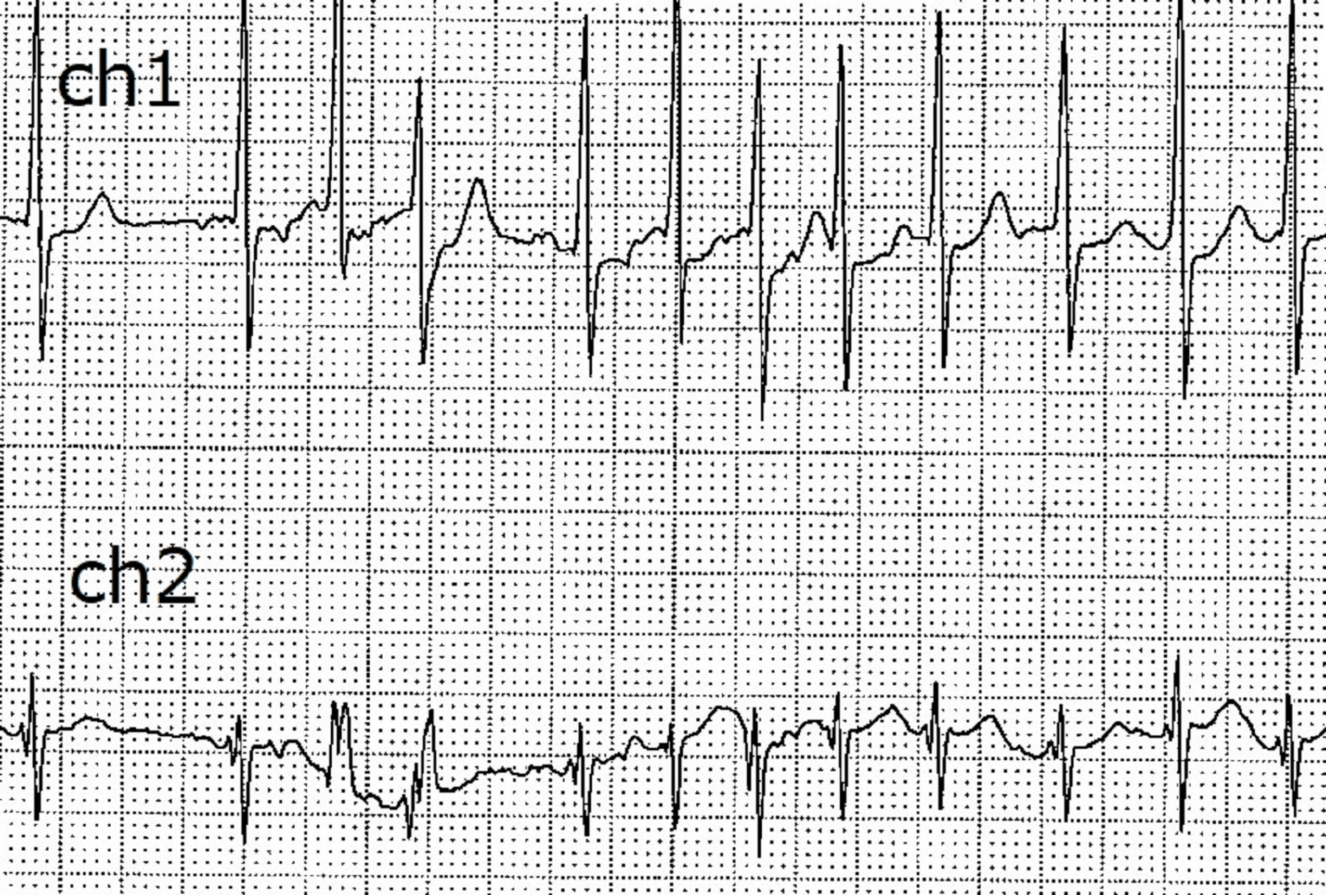
Preoperative Holter electrocardiogram Tachycardia with atrial fibrillation was frequently observed in a day. Fifty-three percent of the total of 148,472 beats per day were atrial arrhythmias. Each small square represents 0.04 seconds horizontally and 0.1 mV vertically, corresponding to 1 mm in the original.

A 66-year-old man was referred to our hospital because of chest pain with paroxysmal atrial fibrillation. Atrial fibrillation was diagnosed by the referring physician using a Holter electrocardiogram (Figure 1).

**Figure 2:**
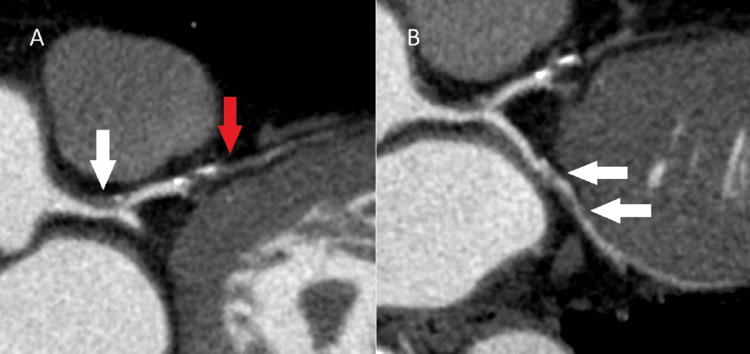
Curved multi-planar reformation of preoperative contrast computed tomography (A) Significant stenosis (white arrow) of the left main trunk and complete occlusion (red arrow) of the left anterior descending artery were suspected. (B) Significant stenosis (white arrows) of the circumflex artery was suspected.

**Figure 3:**
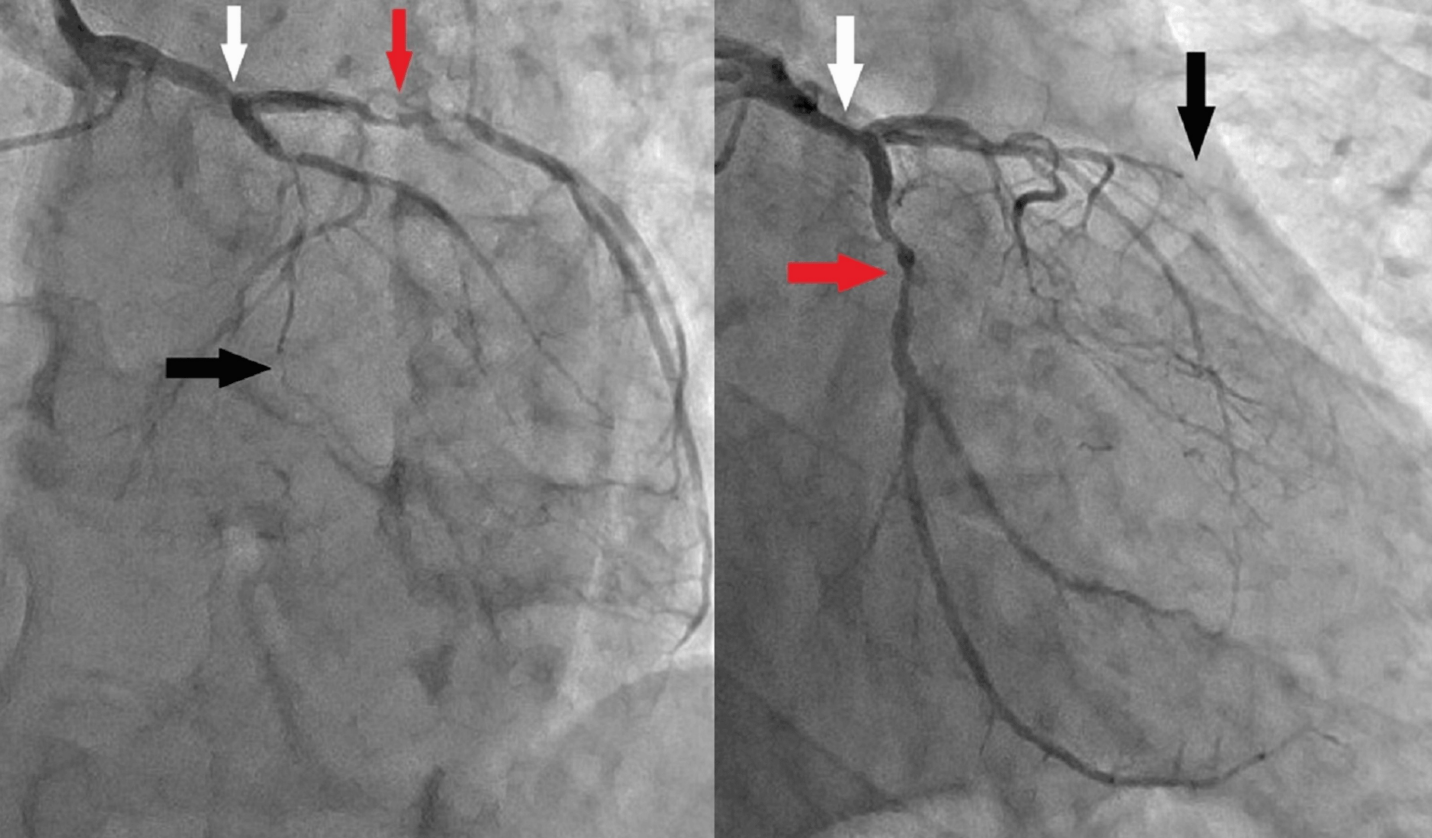
Preoperative coronary angiogram A coronary angiogram revealed 75% stenosis (white arrow) of the left main trunk, complete occlusion (black arrow) of the left descending artery, and 90% stenosis (red arrow) of the circumflex artery.

Fifty-three percent of the 148,472 beats per day were atrial arrhythmia. Bisoprolol fumarate and verapamil had been initiated. The patient’s medical history included dyslipidemia, and he had smoked cigarettes for 20 pack-years until shortly before his hospitalization. Multiple stenoses of the coronary arteries were suspected on contrast CT (Figure 2), and coronary angiography (CAG) revealed 75% stenosis of the left main trunk, complete occlusion of the left anterior descending artery, and 90% stenosis of the circumflex artery (Figure 3).

**Figure 4:**
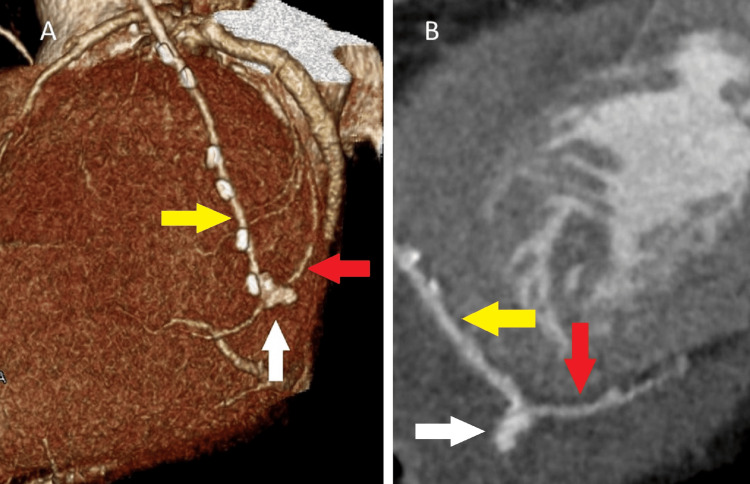
Contrast computed tomography (CT) on postoperative day five A 2.9 mm x 3.1 mm x 8.0 mm pseudoaneurysm (white arrow) was observed at the anastomosis site of the left internal mammary artery (yellow arrow) to the circumflex artery (red arrow). It was visualized only during contrast imaging, while the surrounding hematoma was not enhanced. A: Three-dimensional CT; B: Curved multi-planar reformation

He was still in his 60s and had stenosis of the left main trunk. Atrial fibrillation was paroxysmal. We, the cardiac team, discussed what would be a safer, simpler, and more effective strategy among the combinations of medication, percutaneous coronary intervention, off-pump or on-pump CABG, catheter ablation, operative pulmonary vein isolation (PVI), maze procedure, and left atrium appendage closure. We finally planned the surgical treatment that included PVI using the AtriCure RF ablation system (Century Medical, Inc., Tokyo, Japan), left atrial appendage closure using AtriClip (Century Medical, Inc., Tokyo, Japan), and CABG (right internal mammary artery (RIMA) to left anterior descending artery and LIMA to left circumflex artery anastomoses) without cardiopulmonary bypass (CPB). Both IMAs were skeletonized and anastomosed to the target arteries without tension as we routinely do in young patients [3]. Ventricular tachycardia occurred during PVI; therefore, we performed all procedures under CPB. On-pump beating-heart CABG was performed. The anastomosis sites of the two coronary arteries were only 1.0 - 1.25 mm in diameter. After weaning off CPB, bleeding occurred from the circumflex artery anastomosis. It was controlled using an additional suture, fibrin glue, a TachoSil tissue sealing sheet (CSL Behring, Tokyo, Japan), and manual compression. On postoperative day (POD) 2, after confirming minimal bleeding from the pericardial and mediastinal drains and no progression of anemia, we started heparin at 400 units per hour, warfarin at 2 mg per day, and aspirin at 100 mg per day. The heparin dose was increased to 600 units per hour to achieve an activated clotting time of at least 150 seconds. As a routine post-CABG procedure, contrast CT was performed to verify the patency of the coronary anastomosis on POD 5. It coincidentally revealed a 2.9 mm x 3.1 mm x 8.0 mm outpouching at the anastomosis site of the LIMA to the circumflex artery (Figure 4).

**Figure 5:**
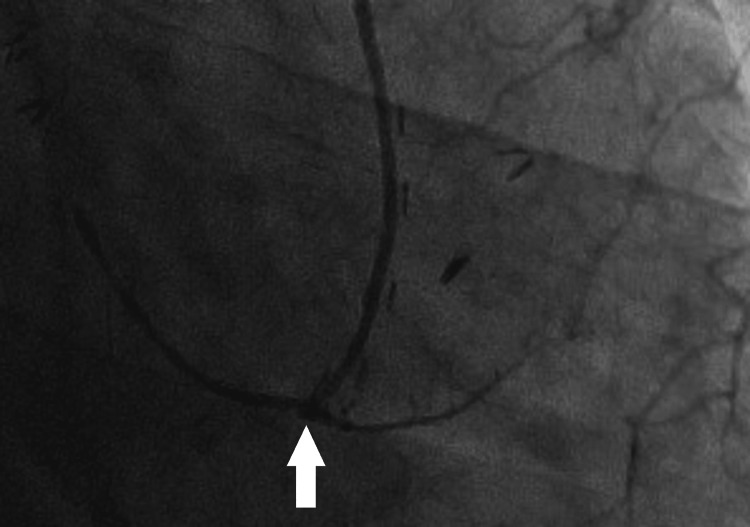
Coronary angiogram performed on postoperative day 13 Contrast was injected into the left internal mammary artery. The image was a frontal view. No pseudoaneurysm (white arrow) was observed, even in other views and when contrast was injected into the left coronary artery.

It was diagnosed as a pseudoaneurysm for the following reasons. A true aneurysm was ruled out because it developed within a short period of time, a clot or hematoma was ruled out because it was only visible with contrast, and a hemorrhage was ruled out because the surrounding hematoma had no contrast effect. Because sinus rhythm persisted after the surgery, warfarin and heparin were discontinued while aspirin was continued after the pseudoaneurysm was found. The pseudoaneurysm was not observed on follow-up CT on POD 8. We also confirmed the resolution of a pseudoaneurysm by another method, CAG (Figure 5).

**Figure 6:**
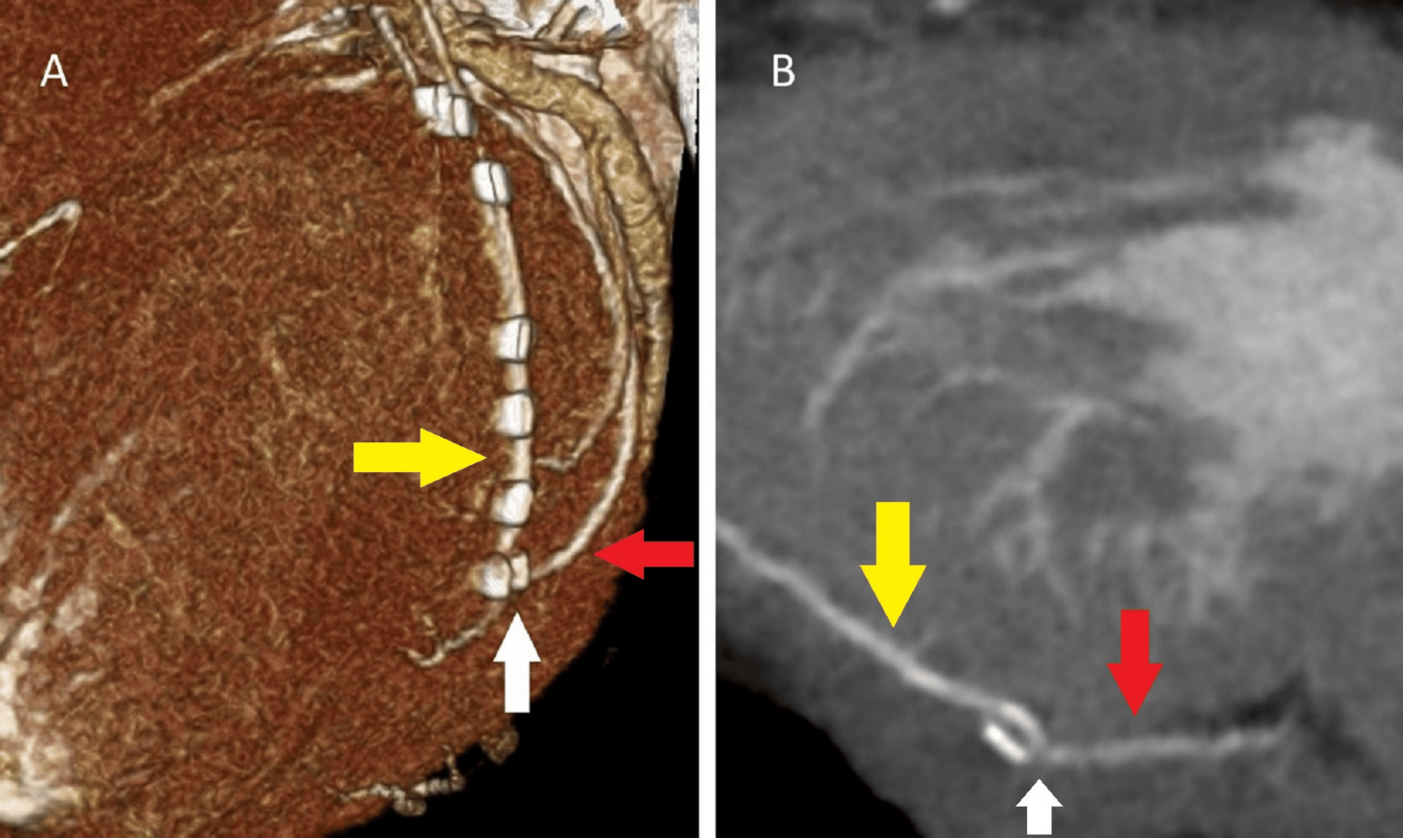
Contrast computed tomography (CT) performed on postoperative day 48 No pseudoaneurysm (white arrow) was observed at the anastomosis site of the left internal mammary artery (yellow arrow) to the circumflex artery (red arrow). A: Three-dimensional CT; B: Curved multi-planar reformation

Therefore, no additional surgery was performed. After discharge from the hospital, a follow-up CT (Figure 6) on POD 48 confirmed the absence of a pseudoaneurysm.

## Discussion

Pseudoaneurysms of the grafts or anastomosis sites after CABG are rare. Previous reports of pseudoaneurysms related to grafts mainly involved saphenous vein grafts, which were treated with surgery or endovascular treatments [1,2]. The natural history of pseudoaneurysms is unclear because they are rare and likely treated early without observation. However, if not diagnosed promptly, it can lead to serious complications [1]. We did not operate on the pseudoaneurysm for three days after it was discovered for the following reasons: 1. It did not rupture, and the size was less than 10 mm; 2. The vital signs were stable. The patient had no symptoms; 3. Spontaneous resolution of pseudoaneurysms detected early after CABG had been reported in three cases [4-6]; 4. Our patient was administered heparin, warfarin, and aspirin when the pseudoaneurysm was discovered. We believed that stopping some of the medication might lead to a resolution.

To the best of our knowledge, only three cases of spontaneous resolution of pseudoaneurysms discovered after CABG have been reported (Table 1). 

**Table 1:** Reports of spontaneous resolution of pseudoaneurysms, including our case CABG: coronary artery bypass grafting; RIMA: right internal mammary artery; LAD: left anterior descending artery; LIMA: left internal mammary artery; PL: posterolateral branch; GEA: gastroepiploic artery; SVG: saphenous vein graft; RCA: right coronary artery; OM: obtuse marginal branch; Cx: circumflex artery; NA: not available; PVI: pulmonary vein isolation; POD: postoperative day

Case	Age	Gender	Procedures	CPB	Site	Size (ｍm）	Finding	Timeline	Antithrombotics
1 [4]	67	Male	CABGx3 (RIMA-LAD, LIMA-PL ,GEA-RCA)	No	RIMA-LAD anastomosis site	NA	POD 10 (incidental)	POD 30: reduction. 6 months: disappearance. 1 year: no recurrence	NA
2 [5]	56	Male	CABGx4 (LIMA-LAD, SVG-RCA, SVG-OM-PL, sequential), Mitral annuloplasty	Yes	SVG-PL anastomosis site	NA	POD 15 (incidental)	3 weeks: disappearance. 6 months: no recurrence	NA
3 [6]	73	Male	CABGx3 (LIMA-LAD, SVG-RCA, SVG-OM)	NA	SVG-RCA graft	15	6 months after surgery (dyspnea)	3 weeks: only remnant. 3.5 months: persistent resolution	Apixaban
4 (our case)	66	Male	CABGx2 (LIMA-Cx, RIMA-LAD), PVI, Closure of left atrial appendage	Yes	LIMA-Cx anastomosis site	2.9 x 3.1 x 8.0	POD 5 (incidental)	POD 8, 13, 48: resolution	Aspirin, heparin, warfarin

Of these, three cases, including our case, are from Japan [4,5]. We suspect that this is because more patients in Japan undergo early postoperative coronary CT or CAG to evaluate grafts than in other countries. In the three reports from Japan, patients were evaluated on POD 15, POD 14, and POD 5, respectively. If more patients worldwide undergo graft evaluation in the early postoperative phase, more pseudoaneurysms and their spontaneous resolution might be reported. These pseudoaneurysms were located at the anastomosis site. Mori et al. [4] identified anastomotic tension as one of the causes of pseudoaneurysm formation. We suspect that the pseudoaneurysm at the anastomosis site may be formed by bleeding from the needle holes with tension or bleeding tendency, and it may heal spontaneously because the holes are small compared to those caused by dislocation of hemoclips. In one report from the United States, a pseudoaneurysm was found approximately six months after surgery [6]. We believe that pseudoaneurysm formation in this reference [6] could be attributed to the use of apixaban for atrial fibrillation. Pseudoaneurysm formation that resolved spontaneously in the literature and in our case may be related to the postoperative coagulopathy induced by CPB, the surgery itself, or antithrombotic medications and resolved with an improvement of the coagulopathy (Table 1).

## Conclusions

We reported a case of spontaneous resolution of a pseudoaneurysm discovered after CABG. Treatment strategies for pseudoaneurysms after CABG include open surgical repair, endovascular intervention, and observation. Due to limited data, there is no consensus on the strategy. However, given the possibility of rupture, surgical or interventional treatment would generally be the first choice. Exceptionally, the findings in this case and previous literature suggest that observation for days to weeks may be one of the management strategies in patients with a small pseudoaneurysm of approximately 10-15 mm or less, stable vital signs, no symptoms, discontinuable antithrombotic medications, a short time after CABG (e.g., three weeks), or a pseudoaneurysm at the anastomosis site. If the observation is chosen as a strategy, strict observation, such as in the intensive care unit, would be required until it is reduced. In the absence of data on the prognosis of resolved pseudoaneurysms, we plan to carefully follow the patient.
